# FGF-2 released from degenerating neurons exerts microglial-induced neuroprotection via FGFR3-ERK signaling pathway

**DOI:** 10.1186/1742-2094-11-76

**Published:** 2014-04-16

**Authors:** Mariko Noda, Kento Takii, Bijay Parajuli, Jun Kawanokuchi, Yoshifumi Sonobe, Hideyuki Takeuchi, Tetsuya Mizuno, Akio Suzumura

**Affiliations:** 1 Department of Neuroimmunology, Research Institute of Environmental Medicine, Nagoya University, Furo-cho, Chikusa-ku, Nagoya 464-8601, Japan; 2 Present address: Department of Anatomy, Keio University School of Medicine, 35 Shinanomachi, Shinjuku-ku, Tokyo 160-8582, Japan

**Keywords:** ERK, FGF-2, FGFR3, microglia, neuroprotection, Wnt

## Abstract

**Background:**

The accumulation of activated microglia is a hallmark of various neurodegenerative diseases. Microglia may have both protective and toxic effects on neurons through the production of various soluble factors, such as chemokines. Indeed, various chemokines mediate the rapid and accurate migration of microglia to lesions. In the zebra fish, another well-known cellular migrating factor is fibroblast growth factor-2 (FGF-2). Although FGF-2 does exist in the mammalian central nervous system (CNS), it is unclear whether FGF-2 influences microglial function.

**Methods:**

The extent of FGF-2 release was determined by ELISA, and the expression of its receptors was examined by immunocytochemistry. The effect of several drug treatments on a neuron and microglia co-culture system was estimated by immunocytochemistry, and the neuronal survival rate was quantified. Microglial phagocytosis was evaluated by immunocytochemistry and quantification, and microglial migration was estimated by fluorescence-activated cell sorting (FACS). Molecular biological analyses, such as Western blotting and promoter assay, were performed to clarify the FGF-2 downstream signaling pathway in microglia.

**Results:**

Fibroblast growth factor-2 is secreted by neurons when damaged by glutamate or oligomeric amyloid β 1-42. FGF-2 enhances microglial migration and phagocytosis of neuronal debris, and is neuroprotective against glutamate toxicity through FGFR3-extracellular signal-regulated kinase (ERK) signaling pathway, which is directly controlled by Wnt signaling in microglia.

**Conclusions:**

FGF-2 secreted from degenerating neurons may act as a ‘help-me’ signal toward microglia by inducing migration and phagocytosis of unwanted debris.

## Background

Neuron and glial cells are in close association with each other and maintain physiological function in the central nervous system (CNS). When their finely controlled interactions are impaired by inflammation and stress conditions, neuronal networks are damaged, which results in the pathogenesis of several neurodegenerative diseases [1-3]. It has been proposed that apoptotic cells or degenerating neurons release various signals to surrounding glial cells. These signals have been recently classified as ‘find-me’, ‘help-me’, and ‘eat-me’ signals [4-8].

Microglia are resident immune cells in the CNS and express many versatile receptors [9]. Therefore, they are considered the main recipient of various signals from degenerating neurons. Moreover, microglia exhibit early and rapid responses to various stimuli; for instance, activated microglia accumulate at pathological lesions [10]. The rapid and accurate migration of microglia to lesions is predominantly mediated by various chemokines [11]. In addition to chemokines, fibroblast growth factor (FGF)-2 regulates cellular migration in developing brain and in zebra fish [12-15]; however, FGF-2 has not been directly implicated in microglial migration.

Fibroblast growth factor, purified from pituitary extracts, has a variety of functions, including inducing the proliferation and differentiation of various cell types, such as fibroblasts. Twenty-two types of FGF have been identified in human beings, as well as in mice. FGF-2 (basic FGF), one of the most common FGFs, has attracted attention for its widespread activity, such as cell proliferation, carcinoma cell invasion, neoangiogenesis, osteogenesis, and differentiation of developmentally staged constituent cells of the CNS [16-19]. FGF-2 is expressed in various tissues at low levels, but its concentration is much higher in the brain. Five types of FGF receptor (FGFR: FGFR1 to 5) have been identified to date [20], but their detailed expression levels in individual cells and mode of action in the CNS have not been elucidated. However, the expression levels of FGF-2 and FGFR have been shown to be up-regulated in CNS injury [21]. Furthermore, several reports show that astrocytes, but not neuronal cells, are the dominant FGF-2-producing cells in the CNS [16-19].

FGF-2 plays important roles in various cells in the CNS. Indeed, morphological change in glial cells and reactivity *in vivo*[22] have been demonstrated with FGF-2 injection into the cerebrospinal fluid. The best known FGF receptor–related signaling is MAPK, which is the common downstream signaling pathway of all FGFR subtypes. FGF-2 is known to induce Wnt/β-catenin signaling in human endothelial cells and developing the zebra fish brain [12,23,24], but it is unclear whether FGF-2 also regulates Wnt/β-catenin signaling in microglia under neurodegenerative conditions.

In this study, we found that FGF-2 was secreted by glutamate or oligomeric amyloid β (oAβ) from damaged neurons, but not from astrocytes or microglia. Degenerating neurons produce signaling molecules that attract surrounding cells including microglia. Among these signaling molecules, we revealed FGF-2 as a predominant coordinator of microglial migration. FGF-2 induced microglial neuroprotection, migration and phagocytosis of neuronal debris via FGFR3. Furthermore, downstream signaling of FGF-2, especially through the FGFR3-extracellular signal-regulated kinase (ERK) signaling pathway, led to microglia-mediated neuronal survival. Wnt signaling directly induced this ERK phosphorylation and microglial migration, which were each enhanced by FGF-2 stimulation. Together, our results demonstrate that FGF-2 could be a key signaling molecule for crosstalk between degenerating neurons and microglia, and that the FGFR3/ERK/Wnt signaling pathway contributes to the induction of microglial neuroprotection.

## Methods

### Reagents

L-glutamate and goat immunoglobulin G (IgG), mouse IgG, and rat IgG were purchased from Sigma (St. Louis, MO, USA). Mouse recombinant FGF-2, mouse recombinant fractalkine (FKN; the chemokine domain), CCL21, and the FGFR (FGFR2-5) neutralizing antibodies were obtained from R & D Systems (Minneapolis, MN, USA). The MAPK inhibitors (U0126 (MEK1/2 inhibitor), SB203580 (p38 inhibitor), and SP600125 (JNK inhibitor)), PI3K inhibitor wortmannin, FGFR antagonist (PD173074 (pan-FGFR blocker), SU11652 (selective FGFR1 blocker)), and IWR-1-endo (Wnt antagonist) were purchased from Calbiochem (Gibbstown, NJ, USA). FGF-2 neutralizing antibody (aFGF-2) was purchased from Millipore (Billerica, MA, USA), and FKN neutralizing antibody (aFKN) was purchased as previously described [25].

### Preparation of Aβ solutions

Aβ1-42 solution was prepared as previously described [26]. Briefly, synthetic human Aβ1-42 (Peptide Institute, Osaka, Japan) was dissolved to 1 mM in 100% 1,1,1,3,3,3-hexafluoro-2-propanol (HFIP). The HFIP was dried and resuspended to a concentration of 5 mM in DMSO. To form oligomers, amyloid peptide was diluted to a final concentration of 100 μM with Ham’s F-12, incubated at 4°C for 24 h, and then immediately added to cultures at a final concentration of 5 μM.

### Cell culture

The protocols for animal experiments were approved by the Animal Experiment Committee of Nagoya University. Primary neuronal cultures were prepared from the cortices of C57BL/6 mice embryos at embryonic day 17 (E17) as described previously [27]. Briefly, cortical fragments were dissociated into single cells in dissociation solution (Sumitomo Bakelite, Akita, Japan), and resuspended in nerve culture medium (Sumitomo Bakelite). Neurons were seeded onto 12 mm polyethylenimine-coated glass coverslips (Asahi Techno Glass Corp., Chiba, Japan). The purity of the cultures was greater than 95%, as determined by NeuN-specific immunostaining [28].

Microglia were isolated from primary mixed glial cell cultures prepared from newborn C57BL/6 mice at day *in vitro* (DIV) 14 using the ‘shaking off’ method, which has been described previously [29]. The purity of the cultures was 97 to 100% as determined by immunostaining for the Fc receptor. Cultures were maintained in DMEM supplemented with 10% fetal calf serum, 5 μg/ml bovine insulin, and 0.2% glucose. Astrocytes were purified from primary mixed glial cultures by three or four repetitions of trypsinization and replating. The purity of astrocytes was greater than 95%, as determined by GFAP-specific immunostaining [30].

### Measurement of FGF-2 levels

Secreted FGF-2 from mouse primary astrocytes, cortical neurons, and microglia were measured using an ELISA kit (RayBiotech, Inc., Norcross, GA, USA). Neurons were treated with L-glutamate (20 μM) or oAβ (5 μM) for 6 to 24 h at 37°C. Supernatants were then collected and assessed for FGF-2 levels.

### Western blotting

Microglial cell lysates were boiled after the addition of sample buffer (1 M Tris-HCl, 20% sodium dodecyl sulfate (SDS), and 2.5% glycerol). Fifty micrograms of total protein were separated on a 5 to 20% Tris-glycine SDS-polyacrylamide gel and blotted onto Hybond-P polyvinylidene difluoride (PVDF) membranes (GE Healthcare UK, Buckinghamshire, UK). Membranes were blocked with 1% skim milk in Tris-buffered saline containing 0.05% Tween 20 for 1 h at room temperature. Primary antibodies to detect phosphorylated and total MAPK (Cell Signaling, Danvers, MA, USA) were applied at the concentrations recommended by the manufacturers. The secondary antibody was horseradish peroxidase-conjugated anti-rabbit IgG (GE Healthcare), which was used at a dilution of 1:1000. SuperSignal West Pico Chemiluminescent Substrate (Thermo Fisher Scientific, Rockford, IL, USA) was used according to the manufacturer’s instructions. The intensities of the bands were calculated using the CS Analyzer 1.0 (Atto Corporation, Tokyo, Japan).

### Wnt promoter assay

HEK293T cells were seeded one day before transfection by FuGENE HD (Promega, Madison, WI, USA) with a luciferase reporter vector from the Cignal TCF/LEF Reporter (luc) kit (Wnt promoter assay system), which was purchased from SABiosciences (Qiagen KK, Tokyo, Japan). After drug treatment, cells were lysed and luciferase reporter activity was measured using the Dual luciferase reporter assay kit (Promega) and a Wallac 1420 ARVOMX (PerkinElmer Japan, Yokohama, Japan).

### Evaluation of microglial phagocytosis

A microglial phagocytosis assay was performed as previously described [25]. Briefly, primary mouse cortical neurons in 24-well plates were labeled on DIV 14 with 1 μM CM-DiI (Molecular Probes), and treated with 20 μM glutamate overnight at 37°C. After changing the culture medium, microglia were added to these neuronal cultures (1:2 ratio for neurons to microglia) with or without FGF-2 for 24 h. Cells were subsequently fixed in 4% paraformaldehyde. Microglia were stained with Cy5-conjugated rat anti-mouse CD11b monoclonal antibodies prior to fixation. Phagocytic uptake of neuronal debris by microglia was estimated based on the detection of DiI-stained neuronal debris [31] in CD11b-positive microglia (green); the phagocytosis index was calculated as the percentage of red staining that overlapped with green staining (shown in yello*w*) among all of the microglia.

### Immunocytochemistry

Cells were fixed with 4% paraformaldehyde, blocked, and permeabilized. Neurons were stained with mouse polyclonal anti–MAP-2 antibody (1:1000; Chemicon, Temecula, CA, USA) and secondary antibody conjugated to Alexa 488 (1:1000; Invitrogen). Astrocytes were stained with mouse monoclonal anti-GFAP antibody (Sigma) and secondary antibody conjugated to Alexa 647 (1:1000; Invitrogen). Microglia were stained with Cy5-conjugated rat anti-mouse CD11b monoclonal antibody (1:300, BD Pharmingen) prior to fixation. Images were analyzed using a deconvolution fluorescence microscope system (BZ-8000; Keyence Corporation, Osaka, Japan). The other primary antibodies included FGFRs, which were purchased from R & D systems and used according to the manufacturer’s instructions.

Surviving neurons were identified based on their cytoskeletons as previously described [28]. Viable neurons were strongly stained with anti-MAP-2 antibodies, whereas damaged neurons showed weaker staining. MAP-2-positive neurons were counted in representative areas in each well. Using five independent trials, more than 200 neurons were evaluated in each well by a scorer who was blind to the experimental conditions. The number of viable neurons in untreated cultures was set as 100%.

### Measurement of CCL3 (MIP-1a), NO, and glutamate levels

Supernatants from microglia were assessed using the chemokine (C-C motif) ligand 3 (CCL3) ELISA kit (R & D Systems), and a Griess reaction for nitric oxide (NO) detection. To measure glutamate levels, a colorimetric assay kit (Yamasa Corporation, Tokyo, Japan) was used, as previously described [25].

### MTS assay

To evaluate the viability of the cells, we used the CellTiter 96 Aqueous One Solution Cell Proliferation Assay kit (Promega) and followed the manufacturer’s instructions.

### Microglial migration assay

Microglial migration was performed using Transwell plates with 3 μm pore polyethylene terephthalate (PET) membrane filters (BD Biosciences). We placed 800 μl of neuronal-conditioned medium or microglial culture medium treated with drugs into the lower chamber of the Transwell plate. Membrane filters were then put in vacant wells, and 200 μl of microglia-containing medium (1.0 × 10^5^ cells/well) was carefully added on top of the filter membrane to avoid bubbles. These plates were incubated for 24 h. Cells that migrated into the lower wells were counted by fluorescence-activated cell sorting (FACS). Chemokine-treated T cells (combination of FKN and CCL21 (100 nM each)) were used as positive controls for this method, as previously described [32].

### RT-PCR

Total RNA was extracted from astrocytes, microglia, and neurons using an RNeasy Mini Kit (Qiagen, Tokyo, Japan). A first-strand cDNA library was obtained using SuperScript II (Invitrogen, Carlsbad, CA) and oligo (dT) 12-18 (Invitrogen) as the first-strand primer. Negative control reactions were performed using the same system after heat denaturation of reverse transcriptase. RT-PCR was used to amplify transcripts encoding mouse FGF-2, each receptor subtypes and glyceraldehydes-3-phosphate dehydrogenase (GAPDH), using 0.1 μg of first-strand cDNA, Blend Taq polymerase (Toyobo Co., Osaka, Japan), and oligonucleotide primers (Table 1; except for previously described primers for GAPDH [25]).

**Table 1:** Oligonucleotide primers used in RT-PCR

**Gene**	**Sequence (5′ to 3′)**	**Expected size (bp)**
FGF-2 sense antisense	5′-AGCGGCTCTACTGCAAGAAC	371
	5′-AGCAGACATTGGAAGAAACAGT	
FGFR1 sense antisense	5′-GTTGGGCTCTGTCATCATCTAT	522
	5′-GCGTACTCCACAATGACATAAA	
FGFR2 (IIIb, IIIc) sense antisense	5′-CTCATCCTGCTGGGTCTGAG	748
	5′-AGGAGTAGCAGCTGATGTGAC	
FGFR3 sense antisense	5′-CCTGTGTAGTTGAGAACAAGTTT	625
	5′-GTGTTGGAGTTCATAGAGGAGT	
FGFR4 sense antisense	5′-GAGGTCTTGTATCTGAGGAACG	651
	5′-GTTCTTGTGTCTTCCGATTAGC	
FGFR5 sense antisense	5′-ATGATATTAGTCCAGGGAAGG	366
	5′-GGATTACATCCACTTTGTAGGT	

### Statistical analysis

Statistically significant differences between experimental groups were determined by one-way analysis of variance (ANOVA) followed by Dunnett’s or Tukey’s tests for multiple comparisons. Statistical analysis was performed using the software program Prism 4 for Windows (GraphPad Software, San Diego, CA, USA). *P* values less than 0.05 were considered significant.

## Results

### Expression of FGFRs in primary neurons and glial cells

**Figure 1:**
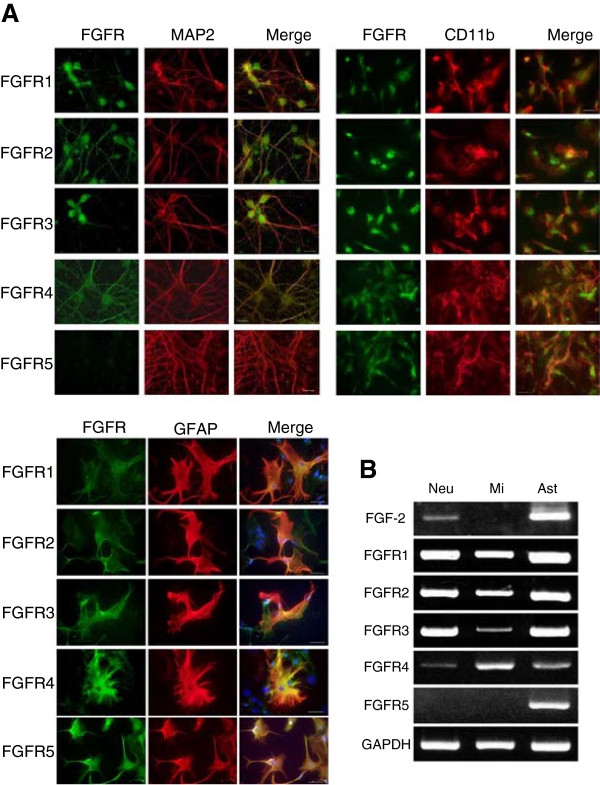
**Expression of FGF-2 and FGFRs in primary neurons and glial cells. (A)** Expression of FGFRs as assessed by immunocytochemistry: FGFRs (green), MAP-2 (mature cortical neurons; red), CD11b (microglia; red), and GFAP (astrocytes; red). Scale bars, 10 μm in neurons and microglia, and 50 μm in astrocytes. **(B)** Expression of FGF-2 and FGFR1 to FGFR5 mRNA in mature cortical neurons (Neu), microglia (Mi) and astrocytes (Ast), as assessed by RT-PCR. GAPDH expression is used as a control.

We first examined the expression of FGFRs in the CNS. According to our immunocytochemical (Figure 1A) and RT-PCR (Figure 1B) data, all FGF receptors (FGFR1 to 5) were expressed in astrocytes. FGFR1 to 4 were expressed in neurons and microglia. The expression of FGF-2 mRNA was detected in neurons and astrocytes.

### Glutamate or oAβ enhances FGF-2 release from neurons, and FGF-2 induces microglial neuroprotection via FGFR3

**Figure 2:**
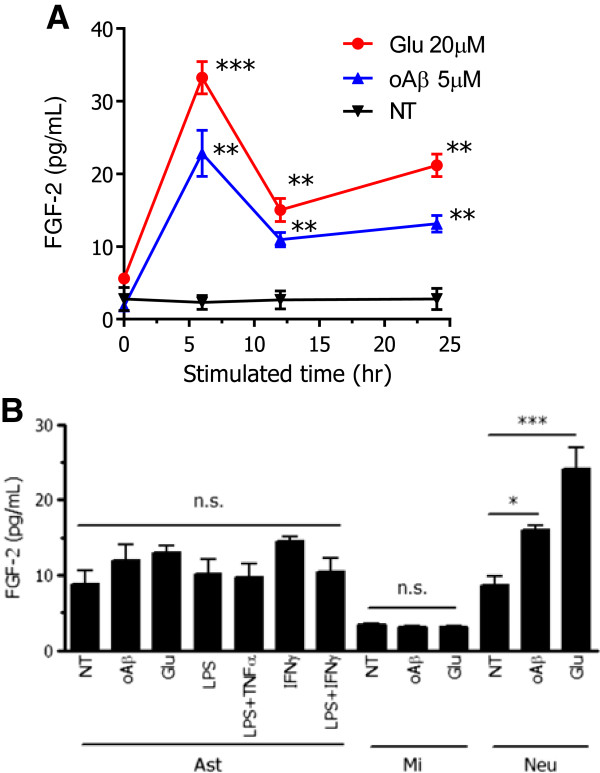
**The detection of FGF-2 in primary neurons and glial cells. (A)** Neurons were treated with glutamate (Glu) or oligomeric amyloid β1-42 (oAβ) at the indicated concentrations. FGF-2 concentrations in the neuronal culture supernatants were measured using ELISAs at each time point. Results show the means with SEM (*n* = 3). Significant differences compared with untreated samples. *: *P* < 0.05, **: *P* < 0.01 (one-way ANOVA with Dunnett’s *post-hoc* test). **(B)** Astrocytes, microglia and neurons were treated with Glu (20 μM) or oAβ (5 μM) for 6 h. Other treatments of astrocytes (6 h) were LPS (1 μg/ml), TNF-α (20 ng/ml), IFN-γ (10 ng/ml), and a combination of all three. ELISA was then performed to detect FGF-2 concentration in the culture supernatants. The results show the means with SEM (*n* = 3). Significant differences compared with untreated samples in each cell type. *: *P* < 0.05, ***: *P* < 0.001, n.s.: not significant (one-way ANOVA with Dunnett’s *post-hoc* test).

FGF-2 is widely expressed in the CNS, especially in astrocytes, while FGF-5, FGF-8, and FGF-9 are synthesized by neurons [33]. FGF-2 is reported to be produced by cerebellar granule neurons in co-cultures with microglia, and to abrogate quinolinic acid–mediated neurotoxicity [31]. In this study, we investigated whether cortical neurons could produce FGF-2 in response to neurotoxic stimuli. We found that treatment for 6 h and 24 h with 20 μM glutamate or 5 μM oAβ significantly induced FGF-2 release from cortical neurons (Figure 2A). Astrocytes typically secrete FGF-2; however, various stimuli including glutamate, oAβ, lipopolysaccharide (LPS), and other proinflammatory cytokines did not enhance FGF-2 secretion by astrocytes (Figure 2B). Furthermore, FGF-2 secretion by microglia was barely detectable (Figure 2B).

**Figure 3:**
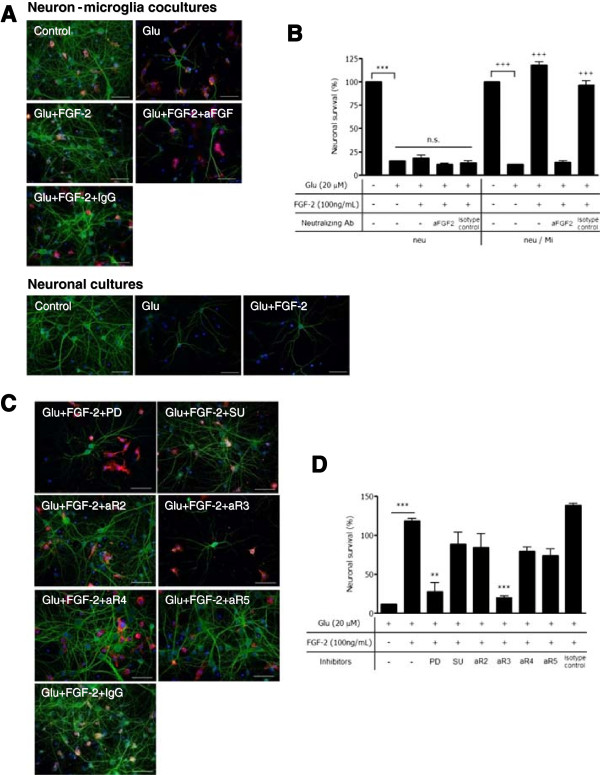
**The neuroprotective effects of FGF-2 in neuron-microglia co-cultures. (A)** Neuronal cultures were also treated with Glu and FGF-2. Neurons were stained with anti-MAP-2 antibody (green), and microglia were stained with a Cy5-conjugated anti-CD11b antibody (red). Scale bars, 50 μm. **(B)** The neuronal survival rate was calculated as the percentage of intact neurons in the treated sample relative to the untreated sample. The columns indicate mean with SEM from three independent experiments. * indicates significant differences compared with untreated neuronal cultures (***: *P* < 0.001); + indicates significant differences compared with untreated neuron–microglia co-cultures (+++: *P* < 0.001) by one-way ANOVA with Tukey’s *post-hoc* test. **(C)** After treatment with 20 μM Glu and 100 ng/ml FGF-2, the inhibitory effects of FGFR were evaluated using FGFR blockers or each anti-FGFR neutralizing antibody (PD, pan-FGFR blocker, 1 μM PD173074; SU, selective FGFR1 blocker, 500 nM SU11652; aR2, anti-FGFR2 antibody; aR3, anti-FGFR3 antibody; aR4, anti-FGFR4 antibody; aR5, anti-FGFR5 antibody; or isotype-matched IgG control). **(D)** The neuronal survival rate was calculated. The columns indicate the means with SEM from three independent experiments, each of which included the analysis of ten randomly selected fields. Significant differences compared with FGF-2-treatment were noted. **: *P* < 0.01, ***: *P* < 0.001 (one-way ANOVA with Tukey’s *post-hoc* test).

Next, we determined whether FGF-2 might exert microglial neuroprotection. As shown in Figure 3A,B, treatment with 20 μM glutamate induced apparent neuronal cell death in neuron-microglia co-cultures. The addition of 100 ng/ml FGF-2 significantly ameliorated neurotoxicity, while an anti-FGF-2 antibody canceled the effect. The addition of rat IgG (isotype-matched control for anti-FGF-2 antibody) had no effect on cell survival rate. In neuronal cultures, neuronal cell death was not ameliorated by FGF-2 treatment. There seems to be little difference in neuronal survival against Glu-induced excitotoxicity with or without microglia. We considered that the secreted level of FGF-2 from Glu-treated neurons might not reach the effective dose to enhance the neuronal survival. In addition, FGF-2 treatment suppressed the proinflammatory response of activated microglia through the inhibition of neurotoxic molecules, such as glutamate and NO (Additional file 1: Figure S1A,B). FGF-2 had no effect on microglial proliferation (Additional file 1: Figure S1C). FGF-2 dose-dependently enhanced the neuronal survival in the presence of microglia (Additional file 1: Figure S2).

To investigate the underlying mechanism of neuroprotection by FGF-2 in microglia, we used FGFR inhibitors and neutralizing antibodies. The neuroprotective effect of 100 ng/ml FGF-2 was completely canceled by treatment with pan-FGFR inhibitor PD173074, or anti-FGFR3 neutralizing antibody. Conversely, neutralizing antibodies for FGFR1, 2, 4, and 5, selective FGFR1 blocker SU11652, and isotype control of neutralizing antibodies had no effect on neuronal survival (Figure 3C,D).

CCL3 (MIP-1α) is reported to be a downstream target of FGF-2-induced FGFR3 signaling [34]. FGF-1-induced FGFR3 targets include the Na^+^ channel, type III intermediate filament peripherin, and cell surface glycoprotein Thy1 [34,35]. We confirmed that FGF-2 leads to the induction of CCL3 expression in microglia. Using ELISA, CCL3 expression was increased by FGF-2 in a dose-dependent manner (Additional file 1: Figure S3). While CCL3 is known as a proinflammatory chemokine, FGF-2 did not activate microglia in this study.

### FGF-2-induced microglial neuroprotection via ERK MAPK and ERK activation is directly regulated by Wnt signaling

**Figure 4:**
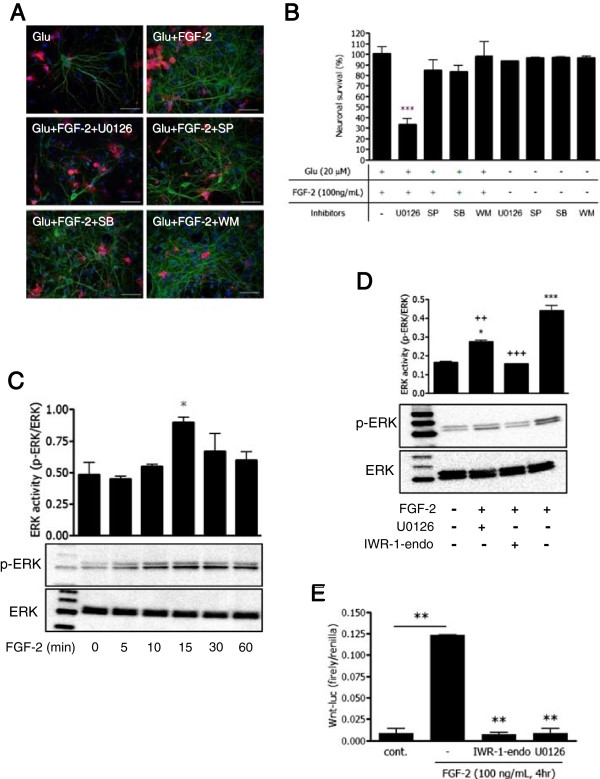
**FGF-2 exhibits neuroprotection via ERK MAPK signaling pathway. (A)** Effects of MAPK and PI3K inhibitors on neuroprotection of FGF-2. U0126, MEK1/2 inhibitor U0126 (1 μM); SP, JNK inhibitor SP600125 (10 μM); SB, p38 inhibitor SB203580 (10 μM); WM, PI3K inhibitor wortmannin (2 μM). **(B)** Neuronal survival rate. Columns indicate mean with SEM from three independent experiments. ***: *P* < 0.001 compared with the cultures without inhibitors (one-way ANOVA with Dunnett’s *post-hoc* test). **(C)** Protein extracts from primary microglia were analyzed by immunoblotting with antibodies specific for phosphorylated and total ERK (ERK1/2). Cells were treated with 100 ng/ml FGF-2 for the indicated periods. *: *P* < 0.05 compared with untreated control (0 min) samples (one-way ANOVA with Dunnett’s *post-hoc* test). **(D)** Microglia were treated with U0126 (1 μM) or Wnt antagonist IWR-1-endo (300 nM) overnight and then 100 ng/ml FGF-2 for 15 min. Western blotting of phosphorylated and total ERK was performed. * indicates significant differences compared with untreated samples (*: *P* < 0.05, ***: *P* < 0.001); + indicates significant differences compared with FGF-2 treatment alone (+++: *P* < 0.001) by one-way ANOVA with Tukey’s *post-hoc* test. **(E)** Wnt promoter assay. HEK293T cells were transfected with Wnt promoter bearing the firefly luciferase reporter vector and Renilla luciferase reporter vector as a transfection control. After 24 h incubation, cells were treated with IWR-1-endo and U0126 overnight, and then treated with FGF-2 for 4 h. Cells were lysed and measured for luciferase activity. **: *P* < 0.01 compared with FGF-2 treatment alone (one-way ANOVA with Dunnett’s *post-hoc* test).

To elucidate the signaling pathway of microglia-mediated neuroprotection, we examined the effect of several kinase inhibitors on neuronal survival. MAPKs (ERK, p38, and JNK) and phosphoinositide-3 kinase (PI3K) are known as common downstream signaling pathways of FGFRs. We found that inhibition of ERK by U0126 significantly suppressed FGF-2-induced microglial neuroprotection. Other kinase inhibitors (p38, JNK, MAPK, and PI3K inhibitors) did not affect neuroprotection (Figure 4A,B). U0126 might affect both microglia and neurons in the co-culture model. The effects of this signaling on neurons cannot be denied. As shown in Figure 4C, FGF-2 increased ERK phosphorylation in microglia, which peaked within 15 min.

In developmental morphogenic stages and angiogenesis, the coordinated action of Wnt/β-catenin and FGF signaling has been reported [23,24,36]. It has also been reported that mouse primary microglia express the Wnt receptors Frizzled and LDL-related protein 5/6 [37]. Therefore, to clarify the interaction of Wnt signaling with FGF in microglia, we examined the effect of Wnt inhibitor on ERK phosphorylation by FGF-2. Pre-treatment of Wnt antagonist IWR-1-endo showed remarkable inhibition of ERK activation (Figure 4D). FGF-2 also directly increased TCF/LEF promoter activity, which is the downstream target of the Wnt signaling pathway. The FGF-2-induced TCF/LEF promoter activity was completely abrogated by treatment of U0126 or IWR-1-endo (Figure 4E).

### FGF-2 increased microglial migration and clearance of neuronal debris via FGFR3 and Wnt pathway signaling

**Figure 5:**
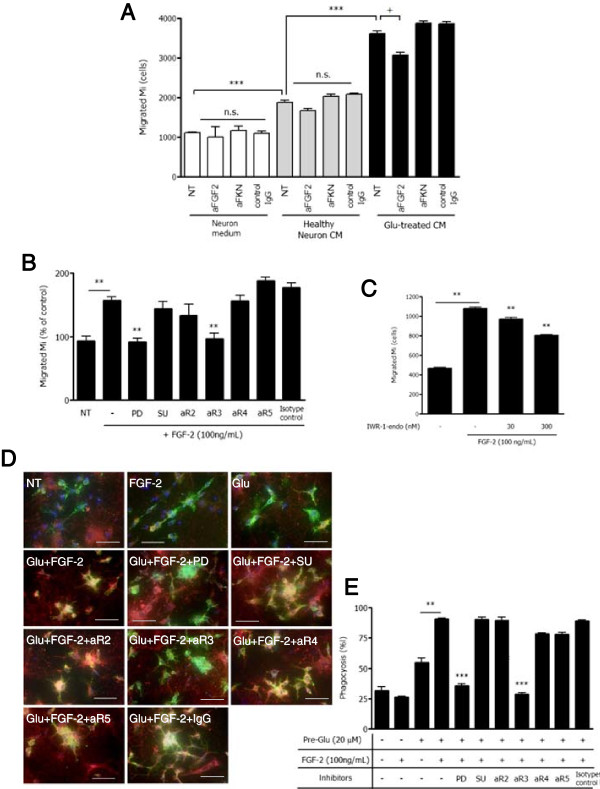
**Effects of FGF-2 on cellular migration and clearance of degenerated neuronal debris by microglia. (A)** Microglial migration assay; aFKN or aFGF-2 were added to lower plates and microglia set on upper Transwell inserts and cultured for 48 h, with isotype-matched IgG as control. Migrated cells in lower plates were counted by FACS. One-way ANOVA with Tukey’s *post-hoc* test: *, significant difference compared with untreated samples using healthy conditioned medium (***: *P* < 0.001); +, significant difference compared with untreated samples (n.s., not significant; +, *P* < 0.05). **(B)** Effect of FGFR blockers and antibodies on microglial migration induced by 100 ng/ml FGF-2. PD, pan-FGFR blocker 1 μM PD173074; SU, selective FGFR1 blocker 500 nM SU11652; aR2, anti-FGFR2 antibody; aR3, anti-FGFR3 antibody; aR4, anti-FGFR4 antibody; aR5, anti-FGFR5 antibody; isotype-matched IgG control. **, *P* < 0.01 compared with FGF-2 treatment alone (one-way ANOVA with Dunnett’s *post-hoc* test). **(C)** Effect of IWR-1-endo on microglial migration induced by FGF-2 treatment. **, *P* < 0.01 compared with FGF-2 treatment alone (one-way ANOVA with Dunnett’s *post-hoc* test). **(D)** Microglial phagocytosis assay. CM-DiI-labeled neurons were treated with or without glutamate, and microglia were added to the culture. These cells were treated with or without 100 ng/ml FGF-2 and with FGFR blockers or each of the anti-FGFR neutralizing antibodies of (B) for 24 h. Scale bars, 20 μm. **(E)** Phagocytosis index, defined as the percentage of total microglia staining (green) overlapping with DiI staining (red). Columns indicate mean with SEM from three independent experiments. Significant differences compared with sample used degenerated neuronal debris and FGF-2 treatment: **, *P* < 0.01; ***, *P* < 0.001 (one-way ANOVA with Tukey’s *post-hoc* test).

We next examined the effect of FGF-2 on microglial migration and phagocytosis activity. We established a microglial migration assay, and assessed migration via the Transwell cell culture system. Microglial migration was significantly increased by CCL21, CCL21 plus FKN, and FGF-2 (Additional file 1: Figure S4). We also confirmed the availability of this system in our previous report [32]. T cells from mouse lymph node showed drastic migration by CCL21 plus FKN (Additional file 1: Figure S4B). Neuronal-conditioned media treated with 20 μM glutamate for 24 h can significantly attract microglia (Additional file 1: Figure S4C). As shown in Figure 5A, while fresh neuronal media did not induce microglial migration, untreated neuronal-conditioned media significantly enhanced migration. Furthermore, it has been determined that neuronal-conditional media treated with 20 μM glutamate for 24 h is a more potent attractant to microglia. This effect was canceled by aFGF-2, but not aFKN (Figure 5A). We also revealed that addition of 100 ng/ml FGF-2 to the lower part of the Transwell system significantly enhanced microglial migration (Figure 5B). The effect was canceled by pan-FGFR inhibitor PD173074 and aFGFR3 neutralizing antibody.

Wnt signaling maintains cell migration in the developmental stages. Therefore we next examined whether Wnt signaling could also mediate microglial migration. Wnt antagonist IWR-1-endo dose-dependently attenuated the induction of microglial migration by FGF-2 (Figure 5C). By contrast, ERK MAPK pathway was not directly concerned with microglial migration (Additional file 1: Figure S4D). Furthermore, FGF-2 enhanced microglial phagocytosis of neuronal debris induced by glutamate toxicity (Figure 5D,E). We examined which type of FGFR is involved in the FGF-2-induced phagocytosis, and found that pan-FGFR inhibitor PD173074 and anti-FGFR3 neutralizing antibody suppressed microglial phagocytosis of neuronal debris (Figure 5D,E).

## Discussion

Our results indicate that FGF-2 is released from degenerating neurons and induces microglial migration and neuroprotection, which are mediated through the FGFR3-Wnt-ERK signaling pathway. Neurons were fine responders of glutamate and oAβ, and then allowed the release of FGF-2 in relatively short times. FGF receptors are expressed in neurons and glial cells. FGFR3, in particular, is activated by FGF-2 via the ERK MAPK-dependent signaling pathway in microglia. The other FGF, FGF-19, is reported to negatively regulate NFκB via FGFR4 [38]. In the developmental morphogenic stages and angiogenesis, coordinated action of Wnt/β-catenin and FGF signaling has been reported [12,23,24,39]. Recently, expression of Wnt receptors Frizzled and LDL receptor-related protein 5/6 has been reported in mouse primary microglia [37]. In this study, we revealed that FGF-2 directly controlled the Wnt signaling pathway in mouse primary microglia, and that Wnt signaling could also directly regulate microglial migration induced by FGF-2. FGF-2 and the extracellular matrix protein Anosmin-1 have dynamic roles in cellular proliferation and migration from the subventricular zone in CNS development [40]. FGF-2 enhances the proliferation and differentiation of neuronal stem cells. Anosmin-1 and FGF-2 could possibly be diagnostic markers in multiple sclerosis (MS), because their expression level varies between different types of MS [16]. In experimental autoimmune encephalomyelitis, the animal model of MS, FGF-2 may act as a remyelinating and nerve fiber preserving agent [41]. Therefore, FGF-2/Wnt signaling has a potential to regulate cellular proliferation and migration to maintain adult CNS function.

Localized delivery of FGF-2 and brain-derived neurotrophic factor (BDNF) to the lesioned hippocampus increases neurogenesis and reduces epileptogenesis in a rat model of epilepsy [42]. The overexpression of FGF-2/BDNF also attenuates neuroinflammation through suppression of IL-1β [43]. Moreover, FGF-2 gene delivery restores hippocampal functions in an Alzheimer’s disease mouse model [44]. FGF-2 has a deep connection with tumorigenicity. CD44-mediated migration of human inflammatory macrophages into the extravascular compartment depends on binding of FGF-2 to the CD44 receptor [45]. Therefore, it is possible that FGF-2 has functional association with a new counterpart other than FGFRs.

The brain concentration of FGF2 is reported to be around 30 to 120 ng/mg [46]; however, some reports show that the concentration is around 50 pg/ml [47,48]. In a future study, we will attempt to clarify the effect of 100 ng/ml FGF2 *in vivo*. Taken together, the present study shows that FGF-2 from damaged neurons functions as help-me and eat-me signals. Targeting the FGF-2/FGFR3 pathway may give us clues for future therapeutic strategy against neurodegenerative diseases.

## Conclusions

The present study shows that FGF-2 could be a key signaling molecule for crosstalk between degenerating neurons and microglia, and the FGFR3/ERK/Wnt signaling pathway in microglia contributes to the induction of neuroprotective function including migration and phagocytosis of neuronal debris. Therefore, FGF-2 from damaged neurons functions as help-me and eat-me signals to microglia.

## Abbreviations

aFGF2: anti-FGF2 neutralizing antibody; aFKN: anti-FKN neutralizing antibody; ANOVA: analysis of variance; BDNF: brain-derived neurotrophic factor; CNS: central nervous system; DIV: day *in vitro*; DMEM: Dulbecco’s modified Eagle medium; DMSO: dimethyl sulfoxide; ELISA: enzyme-linked immunosorbent assay; ERK: extracellular signal-regulated kinase; FACS: fluorescence-activated cell sorting; FKN: fractalkine; FGF: fibroblast growth factor; FGFR: fibroblast growth factor receptor; GAPDH: glyceraldehydes-3-phosphate dehydrogenase; HFIP: 1,1,1,3,3,3-hexafluoro-2-propanol; IgG: immunoglobulin G; LPS: lipopolysaccharide; MS: multiple sclerosis; oAβ: oligomeric amyloid β; PD: pan-FGFR blocker; PET: polyethylene terephthalate; PVDF: polyvinylidene difluoride; RT-PCR: reverse transcriptase polymerase chain reaction; SDS: sodium dodecyl sulfate; SEM: standard error of the mean.

## Competing interests

The authors declare that they have no competing interests.

## Authors’ contributions

MN conducted the ELISAs, microglial phagocytosis assay, FACS analysis, and statistical analysis, and drafted the manuscript. KT performed the RT-PCR experiments and helped draft the manuscript. BP and JK performed the cell culture and were involved in the conception of the study. YS and HT were also involved in the conception of the study. TM carried out the immunocytochemistry and statistical analysis. He was also involved in the conception and design of the study, and helped draft the manuscript. AS was also involved in the conception and design of the study, as well as in preparing the manuscript. All authors read and approved the final manuscript.

## Supplementary Material

Additional file 1: Figure S1 FGF-2 inhibited the release of neurotoxic molecules from activated microglia. **Figure S2.** FGF-2 dose-dependently enhanced neuronal survival in the presence of microglia. **Figure S3.** FGF-2 increased CCL3 (MIP-1α) production in microglia. **Figure S4.** Effects of FGF-2 on microglial migration. Click here for file
