# Supplementary material for: FGF-2 released from degenerating neurons exerts microglial-induced neuroprotection via FGFR3-ERK signaling pathway
Source: J Neuroinflammation. 2014 Apr 16;11:76. doi: 10.1186/1742-2094-11-76 (PMC4022102; doi:10.1186/1742-2094-11-76)
Supplement: Additional file 1: Figure S1 — FGF-2 inhibited the release of neurotoxic molecules from activated microglia. Figure S2. FGF-2 dose-dependently enhanced neuronal survival in the presence of microglia. Figure S3. FGF-2 increased CCL3 (MIP-1α) production in microglia. Figure S4. Effects of FGF-2 on microglial migration. [file 1742-2094-11-76-S1.pdf]

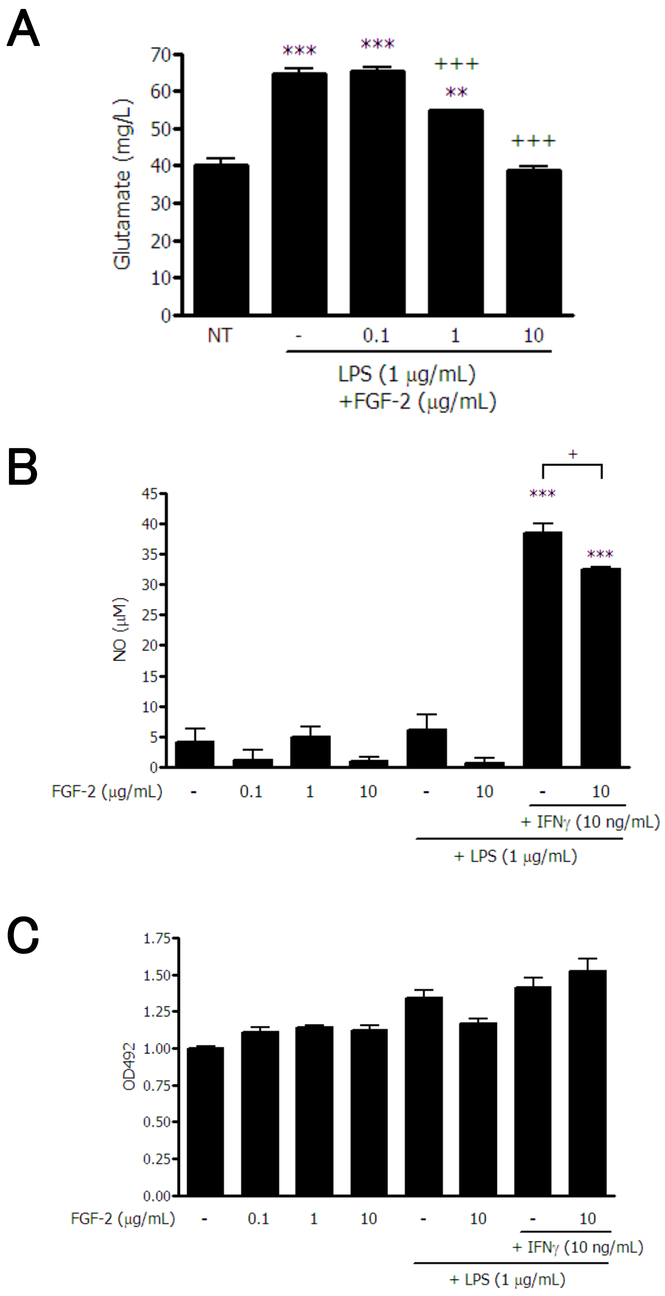

Fig. S1 FGF-2 inhibited the release of neurotoxic molecules from activated microglia

Microglia were treated with FGF-2, LPS (1μg/mL), IFN-γ (10 ng/mL), and their combination for 48 hr. Production of glutamate (A) or NO (B) detected in the culture supernatants. Viability of the cells were assessed using MTS assay (C). Results show the means with S.E.M. (n = 3). * indicates significant differences compared with untreated samples (**: $P < 0.01$, ***: $P < 0.001$); + indicates significant differences compared with treatment LPS alone in *A*, LPS plus IFN-γ in *B* (+: $P < 0.05$, +++: $P < 0.001$).

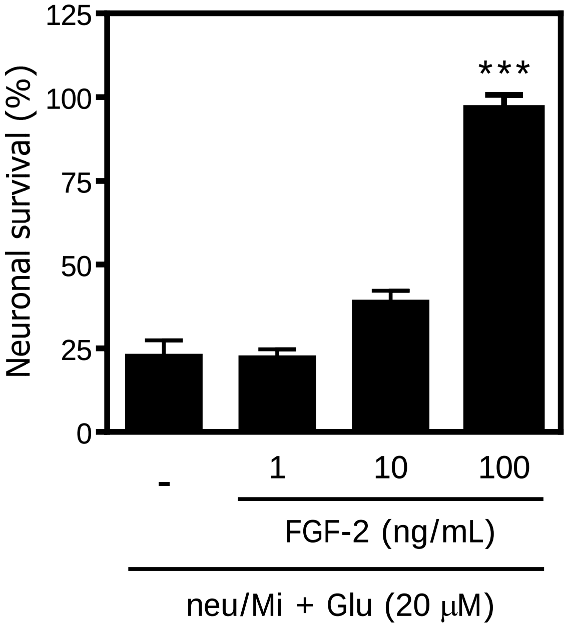

Fig. S2 FGF-2 dose-dependently enhanced neuronal survival in the presence of microglia

Neuron–microglia (1:2) co-cultures were treated with or without 20 μM Glu and indicated concentration of FGF-2 for 24 h. Results show the means with S.E.M. (n = 3). Significant differences compared with untreated controls. ***: P < 0.001 (one-way ANOVA with Dunnett's post-hoc test).

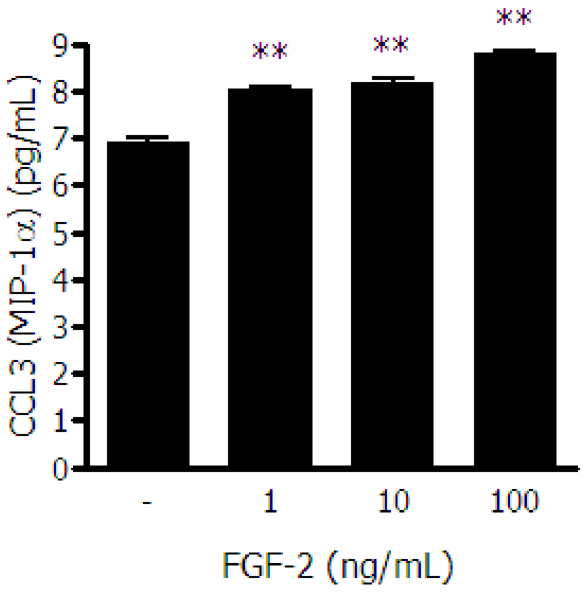

Fig. S3 FGF-2 increased CCL3 (MIP-1α) production in microglia

Microglia were treated with indicated concentrations of FGF-2 for 48 hr. ELISA was performed to detect CCL3 concentration in the culture supernatants. Results show the means with S.E.M. (n = 3). Significant differences compared with untreated controls. **: $P < 0.01$ (one-way ANOVA with Dunnett's post-hoc test).

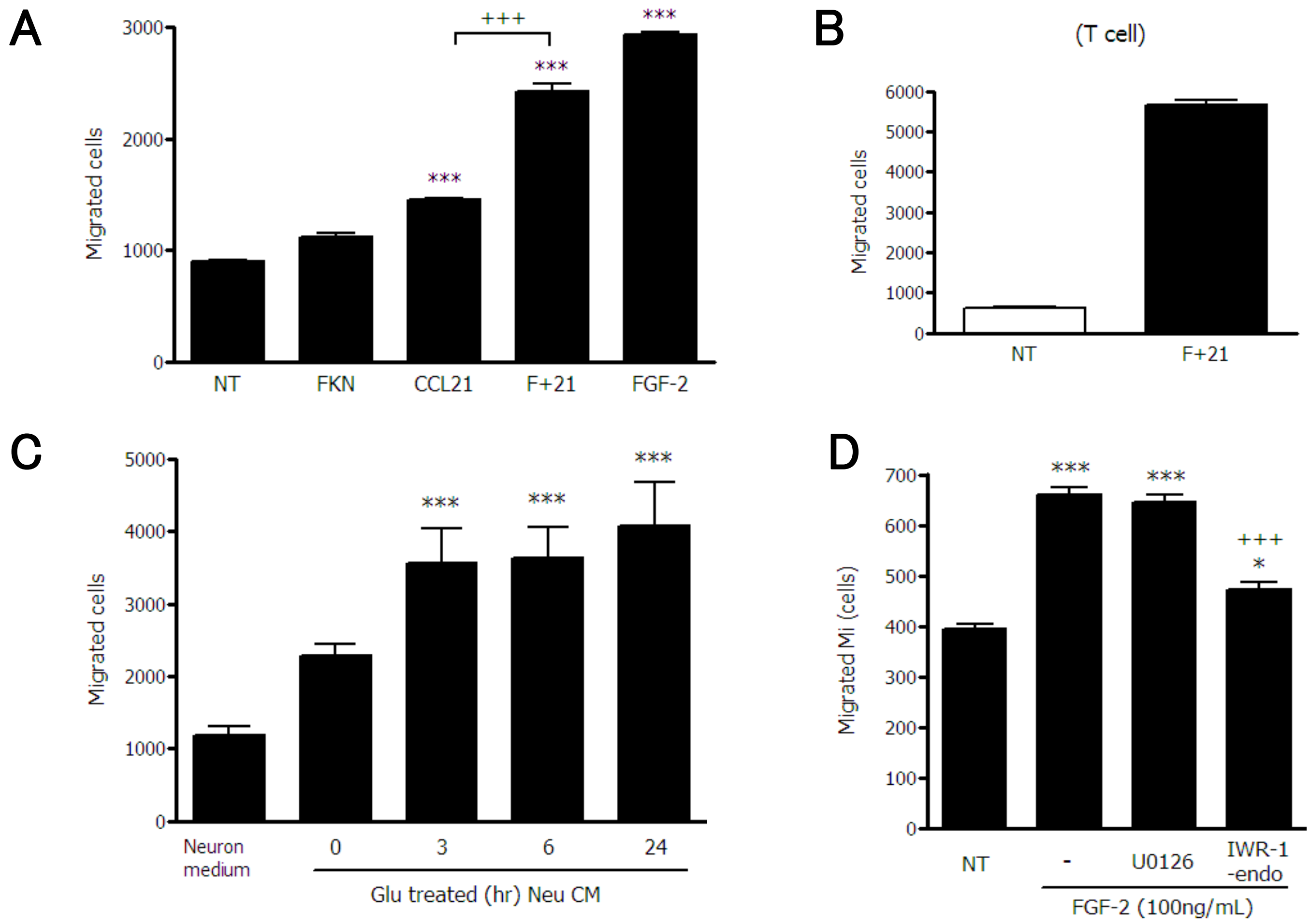

Fig. S4 Effects of FGF-2 on microglial migration

Microglial migration assay was performed using Transwell plates with 3 μm pore sizes PET membrane filters. The filtrated cell number in lower plate was counted by FACS. (A) Microglia were translated to lower plate in the response of chemoattractant for 24 hr (100 nM fractalkine (FKN), 100 nM CCL21, FKN plus CCL21 (F+21) and 100 ng/mL FGF-2). ***: $P < 0.001$ compared with untreated samples, and +++: $P < 0.001$ compared with CCL21 treated samples (one-way ANOVA with Dunnett's post-hoc test). (B) Positive control of this migration assay using T cells placed on to 3 μm pore size membrane filter in Transwell plates. T cells were isolated from mouse lymph node. FKN plus CCL21 (100 nM each) markedly induced the migration. (C) Neuronal conditioned medium treated with Glu (20 μM) for indicated time periods, induces microglial migration in a time-dependent manner. **: $P < 0.01$, ***: $P < 0.001$ compared with neuronal fresh medium (one-way ANOVA with Dunnett's post-hoc test). (D) Effect of U0126 (1 μM) or IWR-1-endo (300 nM) on FGF-2-induced microglial migration. * indicates significant differences compared with untreated samples (***: $P < 0.001$); + indicates significant differences compared with treatment FGF-2 alone (+++: $P < 0.001$) by one-way ANOVA with Tukey's post-hoc test.
